# A latent class analysis of health risk behaviours in the UK Police Service and their associations with mental health and job strain

**DOI:** 10.1186/s12888-022-04054-3

**Published:** 2022-06-24

**Authors:** Patricia Irizar, Suzanne H. Gage, Victoria Fallon, Laura Goodwin

**Affiliations:** 1 School of Social Sciences, Department of Sociology, University of Manchester, Manchester, United Kingdom; 2 The Wellcome Trust, London, United Kingdom; 3 Department of Psychology, Institute of Population Health, University of Liverpool, Liverpool, United Kingdom; 4 The Spectrum Centre for Mental Health Research, Lancaster University, Lancaster, United Kingdom

**Keywords:** Latent class analysis, Post-traumatic stress disorder, Depression, Anxiety, Job strain

## Abstract

**Background:**

Health risk behaviours (e.g., harmful drinking and smoking) often cluster together and can be associated with poor mental health and stress. This study examined how health risk behaviours cluster together in individuals in a high stress occupation (UK Police Service), and the associations with mental health and job strain.

**Methods:**

Data was obtained from the Airwave Health Monitoring Study (25,234 male and 14,989 female police employees), which included measures of health risk behaviours (alcohol use, diet, smoking status, physical activity), poor mental health (depression, anxiety, post-traumatic stress disorder [PTSD]), and job strain (low, high, active, passive). Classes of health risk behaviours were identified using Latent Class Analysis (LCA) and the associations with mental health and job strain were analysed through multinomial logistic regressions.

**Results:**

For men and women, a 5-class solution was the best fit. Men and women with depression, anxiety, and/or PTSD (analysed as separate variables) had at least double the odds of being assigned to the “high health risk behaviours” class, compared to those with no mental health problem. Compared to those reporting low strain, men and women reporting high strain had increased odds of being assigned to the “low risk drinkers with other health risk behaviours” classes.

**Conclusions:**

These finding highlight the importance of holistic interventions which target co-occurring health risk behaviours, to prevent more adverse physical health consequences. Police employees with poor mental health are more likely to engage in multiple health risk behaviours, which suggests they may need additional support. However, as the data was cross-sectional, the temporal associations between the classes and mental health or job strain could not be determined.

**Supplementary Information:**

The online version contains supplementary material available at 10.1186/s12888-022-04054-3.

## Introduction

Health behaviours are actions to maintain, attain or regain good health, such as exercising regularly and eating a balanced diet [1]. Contrarily, health risk behaviours, such as heavy drinking and smoking [2], are major causes of chronic disease and premature mortality [3]. Whilst health risk behaviours are often researched separately, evidence suggests they co-occur [4–6], particularly heavy drinking and smoking [7, 8]. However, not *all* health risk behaviours cluster together. According to the Theory of Triadic Influence (TTI), some behaviours have similar experiences and consequences (e.g., alcohol and nicotine may both be used to alleviate negative affective states), therefore it is logical that they co-occur [9]. Certain sociodemographic characteristics are related to clustered health risk behaviours, such as male gender [7, 10] and older age [11]. Further, clusters of health risk behaviours have associations with mental health problems [12, 13]. One study showed that men and women with depression had double the odds of being assigned to one of the three health risk behaviour clusters, than the healthiest cluster [12]. Additional research identified that individuals reporting more frequent mental distress were more likely to experience clustered poor diet, insufficient physical activity and poor sleep [14].

Work stressors impact health behaviours and mental health [15, 16], with evidence suggesting that high job strain (high demands, low control) is linked to heavy drinking and smoking [17, 18], and poor mental health [19]. High strain could be linked to health risk behaviours through maladaptive coping, but conversely, there is evidence that health behaviours, such as keeping active, can be used as a form of proactive coping, though over-exercising can be problematic [20, 21]. Certain occupations are characterised by stressors (e.g., intensive demands, lack of control) which negatively impact mental health, such as policing [22–24], which could have associations with health risk behaviours. For example, one third of United Kingdom (UK) police employees met criteria for hazardous or harmful drinking, and those with a mental health problem were twice as likely to drink harmfully [25]. Contradicting previous evidence, police employees reporting high strain were less likely to drink hazardously than those with low strain [25], which may reflect proactive coping. These findings highlight the importance of understanding how health risk behaviours cluster in those working in stressful occupations, and to determine the characteristics of those more likely to engage in multiple health risk behaviours.

Policing is a highly stressful occupation and police employees in the UK show high levels of heavy drinking [25]. However, it is not known how health risk behaviours cluster together in UK police employees. Latent Class Analysis (LCA) is a statistical technique used to identify distinct classes based on responses to multiple variables, and has previously been used to determine classes of health risk behaviours in adolescents [26], vocational education students [27], older adults [28] and the UK general population [29]. This study aims to utilise LCA to identify classes of health risk behaviours (alcohol use, fruit and vegetable intake, red meat consumption, smoking, physical activity) in UK police employees, and to determine their associations with mental health (depression, anxiety, PTSD) and job strain (high, low, active, passive). The sociodemographic and occupational associations with the identified classes will be explored, to determine the characteristics of each class and the covariates to be included in the main analyses. This study has been pre-registered with Open Science Framework, where the aims and hypotheses are described in detail https://osf.io/4j7mx.

## Methods

### Study Sample

The study sample was obtained from the Airwave Health Monitoring Study [30]. Data were collected through an enrolment questionnaire sent from administration or occupational health services, and through health screens conducted by trained nurses (advertised through force-wide publicity). Data on a range of health risk behaviours were collected, as well as measures of mental health, job strain, sociodemographics and occupational factors. Data was collected between June 2006 and March 2015 from 28 participating forces (out of 54), with the current sample including 40,986 police officers and staff (response rate averaged 50% across participating forces). The study was open to police employees from all roles (at any level) and police employees could still participate in the health screen even if they did not belong to one of the participating forces. The sample was representative of the UK Police Service in terms of gender and ethnic composition [31]. A detailed design and protocol for the Airwave Health Monitoring Study is available elsewhere [30].

### Measures of health risk behaviours

#### Alcohol use

Two measures of alcohol use were included: categorisation of alcohol consumption (non-drinkers, low risk, hazardous, harmful), and frequency of binge drinking (6 or more drinks on one occasion). Participants who stated “no” to ever drinking alcohol were categorised as “non-drinkers”. The remaining categories were derived from the UK Chief Medical Officer’s guidelines for “low risk” drinking (0–14 units per week) [32], and the National Institute for Health and Care Excellence (NICE) guidelines for hazardous (> 14 to 35/50 units for women/men) and harmful drinking (> 35/50 units for women/men) [33]. Participants stated the number of drinks consumed (white wine, red wine, fortified wine, beer, and spirits) over the past seven days, which was converted to units. Frequency of binge drinking was measured using a single item (“How often do you have six or more drinks on one occasion?”), with the following options: never, monthly or less, two to four times a month, two to three times a week, and daily or almost daily.

#### Smoking status

Smoking status was derived from two items which asked if participants currently smoked cigarettes and if so, how many cigarettes did they smoke per day. Participants were categorised as “non-smokers”, “0 to 10 cigarettes per day” (light to moderate smoking) or “more than 10 cigarettes per day” (heavy smoking) [34].

#### Physical activity

Physical activity was measured using the validated seven-item International Physical Activity Questionnaire – Short Form [35, 36], which has also been validated in occupational settings [37, 38]. The IPAQ-SF measures the frequency, intensity and duration of walking, moderate and vigorous physical activity. Metabolic equivalent (MET; the ratio of work metabolic rate to resting metabolic rate) minutes were computed for each activity (one minute of walking = 3.3 MET mins, moderate activity = 4 MET mins, vigorous activity = 8 MET mins). High physical activity was defined as vigorous intensity activity on at least three days (minimum of 1500 MET minutes), or seven days of any combination of walking, moderate or vigorous intensity activities (minimum of 3000 MET minutes). Moderate physical activity was defined as three or more days of vigorous intensity activity, five or more days of moderate intensity activity, five or more days of walking (at least 30 min per day), or five or more days of any combination of walking, moderate or vigorous intensity activities (minimum of 600 MET minutes per week). Low physical activity was categorised as not meeting either criteria [35]. UK government guidelines recommend achieving a minimum of 600 MET minutes per week [39].

#### Average daily fruit and vegetable intake

Four items measured frequency (days per week) and quantity (how many) of fruit (portions) and vegetable (heaped tbsp.) consumption. Public Health England (PHE) guidance recommends five portions of fruit and vegetables per day, and states that three heaped tbsp of vegetables is equivalent to one portion [40]. Quantity of vegetables was divided by three to reflect portions. Average daily fruit and vegetable intake was computed by calculating total weekly fruit and vegetable intake, multiplying frequency and quantity, then dividing by seven. This was categorised into “2 or less servings per day”, “3 to 4 servings per day”, and “5 or more servings per day”.

#### Red meat consumption

A single item asked, “How often do you eat red meat (beef, veal, lamb, mutton, or pork)?”, with the following response options: “never”, “less than once a week”, “once a week”, “two or more times a week”.

### Measures of mental health and job strain

#### Probable depression

The Patient Health Questionnaire-9 (PHQ-9) [41] is a 9-item self-report screen of symptoms of depression (e.g., trouble sleeping, suicidal thoughts), where responses are provided on a 4-point Likert scale, ranging from “not at all” to “nearly every day”. Scores range from 0 to 27, with a validated cut-off of 10 indicating probable depression. McDonald’s omega (ω) indicated excellent internal reliability within this sample (ω = 0.93).

#### Probable anxiety

The anxiety subscale of the Hospital Depression and Anxiety Scale (HADS-A) [42] is a 7-item self-report screen of symptoms of anxiety (e.g., feeling tense, restlessness). Scores range from 0 to 21, with a validated cut-off of 9 indicating probable anxiety. The scale showed excellent internal reliability within this sample (ω = 0.90).

#### Probable PTSD

The Trauma Screen Questionnaire (TSQ) [43] is a 10-item screen of symptoms of PTSD (e.g., upsetting thoughts/memories, bodily reactions). Participants were only asked these items if they reported a traumatic experience in the 6 months prior to the survey. A 4-point Likert scale was used, ranging from “not at all” to “extremely”. However, responses to the TSQ are usually binary, so “not at all” was coded as 0 and all other response options were coded as 1. Scores ranged from 0 to 10, with a validated cut-off of 6 indicating probable PTSD. The scale showed excellent internal reliability within this sample (ω = 0.99).

#### Job strain

Job strain was measured using six items from the Job Content Questionnaire (JCQ) [44], with four items measuring control and two items measuring demand. The scale showed good internal reliability within this sample (ω = 0.82). A validated quadrant approach was used to group participants into high strain (high demand, low control), low strain (low demand, high control), active strain (high demand, high control), and passive strain (low demand, low control). The sample medians for total scores on the demand items and control items were used to categorise participants into high or low demand and control [45].

### Sociodemographic and occupational measures

The Airwave Health Monitoring Study obtained several self-reported sociodemographic measures: age, gender, country (England, Wales, Scotland), marital status (married/cohabitating, divorced/separated, single, other), education (GSCE or below, vocational qualifications/NVQ1 + 2, A levels/Highers or equivalent, Bachelor’s or postgraduate degree), ethnicity (White, Asian, Black, Mixed ethnic background, other), and number of children under 18 (none, one, two, three or more). Several self-reported occupational variables were measured, including job role (police officer, police staff, other), income (less than £25,999, £26 k to £37,999, £38 k to £59,999, more than £60 k), years in the police force (less than five, six to ten, 11 to 20, more than 20), and number of days sickness absence in the past year (none, one to five, six to ten, more than ten). Body Mass Index (BMI) was derived from participant’s weight and height (underweight > 18.5 kg/m^2^, normal weight 18.5 to 25 kg/m^2^, overweight 25 to 30 kg/m^2^, obese < 30 kg/m^2^).

### Data analysis

**Fig. 1:**
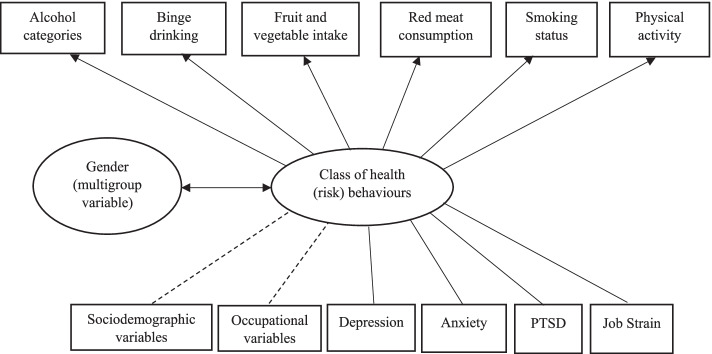
Multigroup latent class model of health (risk) behaviours, grouped by gender, with explanatory variables which were analysed in regression models (sociodemographic variables, occupational variables, mental health and job strain variables)

LCA is a statistical technique used to identify membership in unobserved (latent) subgroups (classes), based on individual responses from multiple variables [46]. For the current study, LCA was conducted in Mplus version 8.3, to determine underlying classes of health risk behaviours by estimating and evaluating a series of models with an incrementally greater number of classes, starting with a 2-class model, to determine the optimal number of classes (Fig. 1).

In LCA, the number of classes is considered optimal when there is homogeneity within the classes and heterogeneity between the classes. The models were evaluated with a range of model fit criteria: Akaike Information Criterion (AIC) [47], Bayseian Information Criteria (BIC) [48], sample size adjusted BIC (SSABIC) [49], entropy values [50], number of bivariate residuals (BVR) [51], and Voung-Lo-Mendell-Rubin likelihood ratio test (VLMR LRT) [52]. Smaller AIC, BIC and SSABIC values indicate better model fit [53]. Larger entropy values (> 0.70) indicate better classification accuracy [50, 54]. Non-significant (*p* > 0.05) VLMR LRT indicates that an additional class does not provide a better fit than a model with one less class, favouring a simpler model [53]. Models containing fewer significant BVR (> 3.84) suggest better model fit [51, 55]. Response probabilities were assessed across all models to determine the most informative and meaningful number of classes [53]. After the optimal number of classes was determined, gender was added using “knownclass”, to examine what the classes looked like for each gender (class memberships and response probabilities can differ for men and women).

Class membership and the conditional probability of assignment to each class were saved in Mplus and imported into STATA SE 15, for the regression analyses. The probability of assignment was used as a probability weight in the regression models to account for individual differences (i.e., individuals in the same class will not all have a probability of 1.0) [56]. Descriptive statistics (mean and standard deviation) explored differences in BMI across the classes.

Unadjusted multinomial regression analyses were first used to determine the sociodemographic (age, education, marital status, children under 18) and occupational (role, years in police force, income, days of sickness absence) associations with each of the classes, separately for men and women (Supplementary materials). Variables with strong statistically significant (*p* < 0.001) associations were included as covariates in the subsequent analyses. Adjusted multinomial regression analyses then determined the mental health (depression, anxiety, PTSD) and job strain (high, low, active, passive) associations with each of the classes, separately for men and women. The reference groups were determined based on size and characteristics of the classes (i.e., sufficient numbers and meaningfulness of reference class).

### Missing data

The proportion of missing data was typically less than 1%, except for PTSD and job role. For PTSD, 6% (*N* = 2,469) of the data was missing (completely at random), as participants who completed the study in 2006 were not provided with the PTSD items. For police role, 9% (*N* = 3,859) of the data was missing, with no record of a result having been received and no explanation why. PTSD and role were only explored as stand-alone variables when looking at their independent associations with the classes of health risk behaviours, and so, the missing data for these variables did not impact the sample size for main latent class model. The minimal missing data for the health risk behaviour variables included in the LCA were handled using Full Information Maximum Likelihood (FIML) estimation, whereby the available data was used to estimate the model and population parameters.

## Results

### Sample characteristics

The sociodemographic and occupational characteristics of the current sample (*N* = 40,986) are outlined in Supplementary table 1. Descriptive statistics for the health risk, mental health, and job strain variables, for the full sample, are shown in Table 1. Most of the sample were low-risk drinkers (55%). Only 14% of the sample reported sufficient fruit and vegetable intake (5 or more servings per day). Around 70% of the sample consumed red meat once a week or more. Almost all participants were non-smokers. Over 50% met criteria for high physical activity. The screening measures of mental health indicated that 10% met criteria for probable depression, 8% for probable anxiety and 4% for probable PTSD.

**Table 1:** Descriptive statistics for health (risk) and mental health variables (*N* = 40,986)

Variable		Complete N / % Missing		N	%	95% CI
*Alcohol*						
Past weekly consumption	40,986 (0.00)					
Non-drinkers			3,764		9.18	8.91 to 9.47
Low risk (0–14 units)			22,612		55.17	54.69 to 55.65
Hazardous drinkers (> 14–35/50 units)			13,365		32.61	32.16 to 33.06
Harmful drinkers (> 35/50 units)			1,245		3.04	2.88 to 3.21
6 or more drinks on one occasion	40,986 (0.00)					
Never			11,187		27.29	26.87 to 27.73
Monthly or less			17,390		42.43	41.95 to 42.91
Two to four times a month			8,469		20.66	20.27 to 21.06
Two to three times a week			3,278		8.00	7.74 to 8.26
Daily/almost daily			662		1.62	1.50 to 1.74
*Diet*						
Fruit & vegetable intake	40,876 (0.27)					
5 or more servings per day			5,664		13.86	13.52 to 14.19
3 to 4 servings per day			11,710		28.65	28.21 to 29.09
2 or less servings per day			23,502		57.50	57.02 to 57.97
Red meat consumption	40,876 (0.27)					
Never			2,184		5.34	5.13 to 5.57
Less than once a week			10,018		24.51	24.09 to 24.93
Once a week			14,100		34.49	34.03 to 34.96
Two or more times a week			14,574		35.65	35.19 to 36.12
*Smoking status*	40,942 (0.11)					
Non-smoker			37,338		91.20	90.92 to 91.47
0 to 10 cigarettes a day			2,403		5.87	5.65 to 6.10
More than 10 cigarettes a day			1,201		2.93	2.77 to 3.10
*Physical activity*	40,978 (0.02)					
High physical activity			20,731		50.59	50.11 to 51.07
Moderate physical activity			14,040		34.26	33.80 to 34.72
Low physical activity			6,207		15.15	14.80 to 15.49
*Body mass index (BMI)*	40,922 (0.16)					
Underweight > 18.5 kg/m^2^			138		0.34	0.29 to 0.40
Normal weight 18.5 to 25 kg/m^2^			12,719		31.08	30.63 to 31.53
Overweight 25 to 30 kg/m^2^			19,042		46.53	46.05 to 47.02
Obese < 30 kg/m^2^			9,023		22.05	21.65 to 22.45
*Mental health*						
Depression	40,372 (1.50)					
Probable case			3,958		9.80	9.52 to 10.10
Non-case			36,414		90.20	89.90 to 90.48
Anxiety	40,372 (1.50)					
Probable case			3,407		8.44	8.17 to 8.71
Non-case			36,965		91.56	91.29 to 91.83
PTSD	38,517 (6.02)					
Probable case			1,520		3.95	3.76 to 4.15
Non-case			36,997		96.05	95.85 to 96.24
Job strain^a^	40,372 (1.50)					
Low			9,722		24.08	23.67 to 24.50
High			11,015		27.28	26.85 to 27.72
Active			11,246		27.86	27.42 to 28.30
Passive			8,389		20.78	20.39 to 21.18

^a^Quadrant approach used to create categories of job strain based on sample medians for demand and control: low (low demands, high control), high (high demands, low control), active (high demands, high control), passive (low demands, low control)

### Latent class analysis of health risk behaviours

The LCA identified a five-class solution as the best fit for the full sample, based on the model fit criteria and meaningfulness of the classes (Table 2). The AIC, BIC and SSABIC decreased with every additional class, though reductions levelled off after five classes (supplementary Fig. 1). The number of significant BVRs decreased from 20 for four classes, to seven for five classes, suggesting greater conditional independence for a five-class solution. Entropy was highest for four classes (0.85), indicating better classification accuracy, though this was still sufficient for five classes (0.71) [54]. When examining the item-response probabilities across models with an incremental number of classes, the additional fifth class was meaningful as it created two distinct classes of varied health and health risk behaviours (i.e., low-risk drinkers but engaged in other health risk behaviours *versus* physically active but engaged in other health risk behaviours). After selecting a five-class model, gender was added using the “knownclass” function, to allow for gender differences in assignment to the classes. The entropy value and number of significant BVRs indicated that a five-class solution was a good fit to the multigroup model.

**Table 2:** Model fit criteria for deciding the number of classes for the full sample (*N* = 40,986), then for the multigroup analysis with gender added using the known class function, to determine number of classes for men (*N* = 25,788) and women (*N* = 15,198). Derived from Mplus

Number of classes	Entropy	AIC	BIC	SSABIC	VLMR-LRT (*p*-value)	BVR	Range of class probabilities
Full sample							
2	0.71	465,395.85	465,680.35	465,575.47	16,062.07 (0.000)	38	0.87 to 0.97
3	0.78	457,361.07	457,792.12	457,633.22	8024.34 (0.000)	25	0.88 to 0.97
4	0.85	456,432.73	457,010.34	456,797.41	957.04 (0.000)	20	0.60 to 0.99
**5**	**0.71**	**455,663.65**	**456,387.81**	**456,120.86**	**798.66 (0.000)**	**7**	**0.60 to 0.86**
6	0.71	455,486.82	456,357.54	456,036.56	209.67 (0.000)	5	0.31 to 0.90
7	0.63	455,363.84	456,381.12	456,006.11	156.12 (0.152)	3	0.29 to 0.92
8	0.64	455,198.67	456,362.50	455,933.47	113.96 (0.001)	2	0.43 to 0.82
Multigroup							
**5**	**0.77**	**505,344.14**	**506,766.60**	**506,242.23**	**-**	**4**	**-**

*AIC* Akaike Information Criterion, *BIC* Bayesian Information Criterion *SSABIC* Sample-size adjusted Bayesian Information Criterion, *VLMR-LRT* Voug-Lo-Mendell-Rubin adjusted Likelihood Ratio Test, *BVR* bivariate residuals

The response probabilities for men (*N* = 25,788) and women (*N* = 15,198) are shown in Table 3 and the class descriptions are defined in Table 4, with the probability-weighted proportions of participants in each class. There were five distinct classes for men and women. Both genders had a “healthiest” class (17.6% of men, 28.7% of women) and a “healthy abstainers” class (13.8% of men, 16.2% of women). These two classes can be distinguished as the “healthiest” class consisted mostly of drinkers (at a low-risk level) and had the highest probabilities for sufficient fruit/vegetable intake, low red meat consumption, non-smoking, and physical activity, whereas the “healthy abstainers” class comprised mostly abstainers who also had high probabilities (but not as high) for these items. The largest class for both genders was “low risk drinkers but other health risk behaviours” (35.6% of men, 39.1% of women), with high probabilities of low-risk drinking, but also insufficient fruit/vegetable intake and low physical activity. The smallest class for both genders was “high health risk behaviours” (4.1% of men, 3.8% of women). For men, the final class reflected “some health risk behaviours but physically active” (28.9%), as this class was defined by hazardous/binge drinking, light/moderate smoking, but high physical activity. For women, the final class reflected “moderate health risk” (12.2%), which had similar characteristics to the final class for men.

**Table 3:** Class probabilities for each class, by gender. The largest probabilities for each class are bolded and the smallest probabilities for each class are italicised. Derived from Mplus

	Men *N* = 25,788					Women *N* = 15,198				
Variable	Class 1 *N* = 5,407 (17.59%)	Class 2 *N* = 3,560 (13.79%)	Class 3 *N* = 7,092 (28.86%)	Class 4 *N* = 8,839 (35.62%)	Class 5 *N* = 990 (4.14%)	Class 1 *N* = 4,543 (28.72%)	Class 2 *N* = 1,894 (16.22%)	Class 3 *N* = 2,072 (12.19%)	Class 4 *N* = 6,121 (39.07%)	Class 5 *N* = 568 (3.80%)
Alcohol										
Past weekly consumption										
Non-drinkers	*0.00*	**0.55**	*0.00*	*0.00*	*0.00*	*0.00*	**0.72**	*0.00*	*0.00*	*0.00*
Low risk	**0.75**	0.44	0.04	**0.77**	0.03	**0.89**	0.28	0.20	**0.89**	0.06
Hazardous drinkers	0.25	0.01	**0.93**	0.23	0.42	0.11	*0.00*	**0.77**	0.11	**0.55**
Harmful drinkers	*0.00*	*0.00*	0.03	*0.00*	**0.55**	*0.00*	*0.00*	0.03	*0.00*	0.40
6 or more drinks on one occasion										
Never	0.17	**1.00**	*0.00*	0.15	*0.01*	0.37	**1.00**	*0.00*	0.26	*0.00*
Monthly or less	**0.68**	*0.00*	0.20	**0.71**	0.03	**0.55**	*0.00*	0.31	**0.61**	0.09
Two to four times a month	0.14	*0.00*	**0.54**	0.14	0.12	0.08	*0.00*	**0.56**	0.12	0.32
Two to three times a week	0.01	*0.00*	0.25	0.01	**0.46**	0.01	*0.00*	0.13	0.01	**0.45**
Daily/almost daily	*0.00*	*0.00*	0.01	*0.00*	0.38	*0.00*	*0.00*	*0.00*	*0.00*	0.14
Diet										
Fruit & vegetable intake										
2 or less servings per day	*0.00*	**0.57**	**0.61**	**0.84**	**0.71**	*0.20*	**0.53**	**0.50**	**0.84**	**0.67**
3 to 4 servings per day	**0.59**	0.27	0.28	0.15	0.19	**0.53**	0.29	0.35	0.12	0.23
5 or more servings per day	0.41	*0.17*	*0.12*	*0.01*	*0.10*	0.26	*0.18*	0.15	*0.04*	*0.10*
Red meat consumption										
Never	*0.03*	*0.07*	*0.01*	*0.02*	*0.01*	*0.13*	*0.15*	*0.07*	*0.07*	*0.06*
Less than once a week	0.24	0.26	0.18	0.24	0.15	0.29	0.29	0.27	0.30	0.24
Once a week	0.36	0.32	0.36	**0.38**	0.25	**0.32**	**0.30**	**0.34**	**0.35**	0.35
Two or more times a week	**0.37**	**0.35**	**0.45**	**0.37**	**0.59**	0.26	0.26	0.32	0.28	**0.35**
Smoking status										
Non-smoker	**0.97**	**0.94**	**0.90**	**0.93**	**0.83**	**0.96**	**0.91**	**0.82**	**0.88**	**0.74**
0 to 10 cigarettes	0.02	0.04	0.07	0.05	*0.08*	0.04	0.05	0.14	0.09	0.13
More than 10 cigarettes	*0.01*	*0.03*	*0.03*	*0.03*	0.09	*0.00*	*0.03*	*0.04*	*0.04*	*0.12*
Physical activity										
Low physical activity	*0.05*	*0.15*	*0.11*	*0.19*	*0.17*	*0.11*	*0.19*	*0.15*	*0.25*	*0.21*
Moderate physical activity	0.25	0.29	0.34	0.35	0.35	0.36	0.35	0.38	**0.40**	**0.40**
High physical activity	**0.70**	**0.56**	**0.55**	**0.46**	**0.49**	**0.54**	**0.47**	**0.48**	0.36	0.39

Class descriptions for men: class 1 (healthiest), class 2 (healthy abstainers), class 3 (some health risk behaviours but physically active), class 4 (low risk drinkers but other health risk behaviours), class 5 (highest health risk behaviours). Class descriptions for women: class 1 (healthiest), class 2 (healthy abstainers), class 3 (moderate health risk behaviours), class 4 (low risk drinkers but other health risk behaviours), class 5 (highest health risk behaviours). Class proportions are weighted (using class probability weights)

**Table 4:** Class descriptions and weighted class proportions for each class, separately for men and women, with mean body mass index (BMI) for each class. Class proportions are weighted using class probability weights, derived from Mplus

Class descriptions	
**Men (*N* = 25,788)**	
Class 1: healthiest (17.59%) *Mean BMI = 27.50 kg/m*^*2*^ *(± 3.90)*	Mostly low risk drinkers, who reported rarely binge drinking. Highest fruit and vegetable intake and moderate red meat consumption. Most of this class were non-smokers and reported high physical activity
Class 2: healthy abstainers (13.79%) *Mean BMI = 27.86 kg/m*^*2*^ *(± 4.14) *	Mostly abstainers and low risk drinkers. This class had the second highest fruit and vegetable intake and the second lowest red meat consumption. Mostly non-smokers, with high physical activity
Class 3: some health risk behaviours but physically active (28.86%) *Mean BMI* = *28.11 kg/m*^*2*^* (*± *3.82)*	Mostly hazardous drinkers, who reported binge drinking at least once a week. Average fruit and vegetable intake, with the second highest red meat consumption. This class had the second highest probability of being light to moderate smokers, but reported high physical activity
Class 4: low risk drinkers but other health risk behaviours (35.62%) *Mean BMI* = *27.77 kg/m*^*2*^* (*± *3.94)*	Mostly low risk drinkers who rarely binge drink. This class reported the lowest fruit and vegetable intake and moderate red meat consumption. This class were mostly non-smokers but reported the lowest physical activity
Class 5: high health risk behaviours (4.14%) *Mean BMI* = *29.00 kg/m*^*2*^* (*± *3.84)*	Mostly hazardous and harmful drinkers who reported binge drinking multiple times per week. This class reported very low fruit and vegetable intake and the highest red meat consumption. This class had the highest probability of reporting smoking (light to moderate, and heavy). This class reported the second lowest physical activity
**Women (N = 15,198)**	
Class 1: healthiest (28.72%) *Mean BMI* = *25.97 kg/m*^*2*^* (*± *4.76)*	Mostly low risk drinkers, who reported binge drinking monthly or less. This class reported the highest fruit and vegetable consumption and low red meat consumption. Highest probability of non-smoking and high physical activity
Class 2: healthy abstainers (16.22%) *Mean BMI* = *26.63 kg/m*^*2*^* (*± *5.65)*	Mostly abstainers and low risk drinkers. This class reported moderate fruit and vegetable intake and the lowest red meat consumption. This class were mostly non-smokers, with moderately high physical activity
Class 3: moderate health risk behaviours (12.19%) *Mean BMI* = *26.13 kg/m*^*2*^* (*± *4.33)*	Mostly hazardous drinkers who reported binge drinking at least once a week. Moderate fruit and vegetable intake, and red meat consumption. This class had the highest probability of reporting light to moderate smoking (0–10 cigarettes) but reported high physical activity
Class 4: low risk drinkers but other health risk behaviours (39.07%) *Mean BMI* = *25.75 kg/m*^*2*^* (*± *4.85)*	Mostly low risk drinkers who reported rarely binge drinking. This class reported the lowest fruit and vegetable intake and high red meat consumption. This class included some light to moderate smokers and reported the lowest physical activity
Class 5: high health risk behaviours (3.80%) *Mean BMI* = *27.00 kg/m*^*2*^* (*± *4.87)*	Mostly hazardous and harmful drinkers, who reported binge drinking multiple times per week. Low fruit and vegetable intake and the highest red meat consumption. This class had some light to moderate, and heavy, smokers. This class reported the second lowest physical activity

### Sociodemographic and occupational associations

The sociodemographic and occupational associations with each class are outlined in Supplementary table 2 for men and Supplementary table 3 for women. The “healthiest” class was the reference group for both men and women.

Men over 40 had double the odds of being in the “high health risk” class, and men aged over 50 had 1.5 times greater odds of being in the “healthy abstainers” class, but reduced odds of being in the “low risk drinkers but other health risks” class (versus < 29 years old). Men who held a GCSE education or below had increased odds of being in the “high health risk” class, compared to all other categories. Asian men showed 9 times greater odds of being in the “healthy abstainers” class, and Black men showed 3 times greater odds (versus White men). Police staff showed 1.7 times greater odds of being in the “healthy abstainers” class than police officers, and those who had served for over 10 years (versus < 5) showed double the odds of being in the “high health risk” class. Men with the lowest income (versus all other categories) were significantly more likely to be in the “healthy abstainers” class. Men with more than 10 days of sickness absence (versus none) had at least 1.5 times greater odds of being in the “high health risk” class and the “healthy abstainers” class.

Women aged 40–49 years old had double the odds of being in the “high health risk” class, and women over 50 had reduced odds of being in the “moderate health risk” class (versus < 29 years old). Women under 29 had increased odds of being in the “low risk drinkers but other risks” class, versus all other age groups. Women with a bachelor’s/postgraduate degree showed reduced odds of being assigned to the “high health risk” and “low risk drinkers but other risks” classes (versus GCSE education or below). Asian women had 5.4 times greater odds of being in the “healthy abstainers” class, and Black women had 4.6 times greater odds, compared to White women. Single women had at least 1.3 times greater odds of being assigned to the “low risk drinkers but other risks” and “moderate health risk” classes, than married/cohabiting women. Women who had served for more than 10 years (versus < 5) showed 1.8 times greater odds of being in the “high health risk” class, but reduced odds of being in the “low risk drinkers but other risks” class. Women with more than 10 days of sickness absence (versus none) were 1.6 times more likely to be in the “high health risk” class and “healthy abstainers” class.

### Mental health and job strain associations

The mental health and job strain associations with class membership are shown in Table 5, separately for men and women. The sociodemographic and occupational variables found to be significantly associated with class membership in men and women (age, country, education, ethnicity, marital status, number of children under 18, years in police force, income, days of sickness absence in past year) were included as covariates (except for role, due to a large proportion of missing data). The “healthiest” classes for men and women were the reference groups.

**Table 5:** Multinomial logistic regressions examining the mental health and job strain associations with the identified classes of health (risk) behaviours for men and women. Percentages are weighted with conditional probability weights. Adjusted multinomial odds ratios (AMOR), with 95% confidence intervals (CIs) are shown). Adjusted for sociodemographic and occupational variables found to be significantly associated with the identified classes for men and women (age, country, education, ethnicity, marital status, number of children under 18, years in police force, income, days of sickness absence in past year)

	**Class 1 N = 5,307** **Healthiest (Ref.)**	**Class 2 N = 3,560** **Healthy abstainers**		**Class 3 N = 7,092** **Health risks but physically active**		**Class 4 N = 8,839** **Low risk drinkers but other risks**		**Class 5 N = 990** **High health risk behaviours**	
**Men (*N* = 25,788)**	**N (%)**	**N (%)**	**AMOR (95% CI)**	**N (%)**	**AMOR (95% CI)**	**N (%)**	**AMOR (95% CI)**	**N (%)**	**AMOR (95% CI)**
Depression									
Non-case	4,981 (95.07)	3,163 (89.35)	1.00	6,381 (91.86)	1.00	7,928 (91.11)	1.00	830 (85.40)	1.00
Probable case	257 (4.93)	348 (10.65)	2.07 (1.73 to 2.47)***	573 (8.14)	1.66 (1.42 to 1.95)***	775 (8.89)	1.80 (1.55 to 2.10)***	137 (14.60)	3.09 (2.45 to 3.89)***
Anxiety									
Non-case	5,030 (96.16)	3,244 (92.00)	1.00	6,476 (93.14)	1.00	8,200 (94.34)	1.00	845 (87.09)	1.00
Probable case	208 (3.84)	267 (8.00)	2.10 (1.72 to 2.56)***	478 (6.86)	1.82 (1.53 to 2.16)***	503 (5.66)	1.49 (1.25 to 1.77)***	122 (12.91)	3.51 (2.75 to 4.48)***
PTSD									
Non-case	4,773 (96.68)	3,228 (95.46)	1.00	6,273 (95.56)	1.00	8,076 (96.43)	1.00	818 (91.80)	1.00
Probable case	164 (3.32)	145 (4.54)	1.34 (1.04 to 1.71)*	291 (4.44)	1.34 (1.09 to 1.64)**	306 (3.57)	1.09 (0.89 to 1.33)	73 (8.20)	2.53 (1.89 to 3.40)***
Job Strain									
Low	1,598 (21.78)	990 (27.95)	1.00	2,137 (30.99)	1.00	2,484 (28.18)	1.00	306 (32.27)	1.00
High	1,142 (21.78)	878 (25.79)	1.21 (1.07 to 1.38)**	1,491 (21.52)	1.00 (0.89 to 1.11)	2,080 (24.40)	1.14 (1.03 to 1.26)**	208 (21.65)	1.01 (0.82 to 1.23)
Active	1,525 (29.45)	1,018 (28.78)	1.10 (0.98 to 1.24)	2,120 (30.29)	1.04 (0.94 to 1.14)	2,484 (28.47)	1.07 (0.97 to 1.17)	303 (30.81)	1.05 (0.88 to 1.26)
Passive	973 (18.53)	625 (17.47)	0.95 (0.83 to 1.09)	1,206 (17.20)	0.92 (0.82 to 1.03)	1,651 (18.95)	1.03 (0.93 to 1.15)	150 (15.26)	0.83 (0.67 to 1.03)
	**Class 1 N = 4,543** **Healthiest (Ref.)**	**Class 2 N = 1,894** **Healthy abstainers**		**Class 3 N = 2,072** **Moderate health risk behaviours**		**Class 4 N = 6,121** **Low risk drinkers but other risks**		**Class 5 N = 568** **High health risk behaviours**	
**Women (*N* = 15,198)**	**N (%)**	**N (%)**	**AMOR (95% CI)**	**N (%)**	**AMOR (95% CI)**	**N (%)**	**AMOR (95% CI)**	**N (%)**	**AMOR (95% CI)**
Depression									
Non-case	4,034 (90.22)	1,612 (86.25)	1.00	1,612 (87.47)	1.00	5,280 (86.86)	1.00	429 (72.98)	1.00
Probable case	439 (9.78)	258 (13.75)	1.36 (1.14 to 1.62)**	255 (12.53)	1.31 (1.10 to 1.55)**	775 (13.14)	1.39 (1.22 to 1.58)***	141 (27.02)	3.24 (2.57 to 4.08)***
Anxiety									
Non-case	3,982 (89.02)	1,637 (87.72)	1.00	1,784 (86.98)	1.00	5,319 (87.91)	1.00	448 (79.30)	1.00
Probable case	491 (10.98)	233 (12.28)	1.10 (0.92 to 1.31)	256 (13.02)	1.20 (1.01 to 1.42)*	736 (12.09)	1.14 (1.01 to 1.30)*	113 (20.70)	2.16 (1.70 to 2.75)***
PTSD									
Non-case	4,140 (96.58)	1,654 (96.39)	1.00	1,677 (95.54)	1.00	5,681 (96.39)	1.00	518 (93.54)	1.00
Probable case	147 (3.42)	63 (3.61)	1.01 (0.74 to 1.37)	80 (4.46)	1.35 (1.02 to 1.80)*	213 (3.61)	1.09 (0.87 to 1.36)	33 (6.46)	1.71 (1.12 to 2.60)*
Job Strain									
Low	1,086 (24.27)	418 (22.70)	1.00	488 (23.78)	1.00	1,370 (22.26)	1.00	138 (24.22)	1.00
High	1,091 (24.40)	508 (26.96)	1.08 (0.92 to 1.27)	496 (24.50)	0.99 (0.85 to 1.16)	1,684 (28.14)	1.19 (1.06 to 1.34)**	144 (25.62)	1.01 (0.78 to 1.32)
Active	1,198 (26.70)	466 (24.93)	1.00 (0.85 to 1.17)	552 (26.70)	1.02 (0.87 to 1.19)	1,452 (23.88)	1.02 (0.91 to 1.14)	124 (22.19)	0.78 (0.59 to 1.02)
Passive	1,098 (24.63)	478 (25.41)	1.04 (0.88 to 1.21)	504 (25.02)	1.02 (0.88 to 1.20)	1,549 (25.72)	1.10 (0.98 to 1.23)	155 (27.97)	1.11 (0.86 to 1.44)

^***^p < 0.001, **p < 0.01, *p < 0.05

Men with probable depression, anxiety or PTSD had 2.5 to 3.5 times greater odds of being in the “high health risk behaviours” class (versus those with no mental health problem). Men with depression or anxiety had double the odds of being in the “healthy abstainers” class, with weaker associations for PTSD. Men with depression, anxiety or PTSD had between 1.3 to 1.8 greater odds of being assigned to the “health risk behaviours but physically active” class. Men with probable depression or anxiety, but not PTSD, had at least 1.5 times the odds of being in the “low risk drinkers but other health risk behaviours” class.

Women with probable depression or anxiety had at least double the odds of being in the “high health risk behaviours” class, compared to those without depression or anxiety. Probable PTSD was also associated with 1.7 times greater odds of being in the “high health risk behaviours” class, and 1.4 times greater odds of being in the “moderate health risk behaviours” class. Women with probable depression had 1.3 times greater odds of being in the “healthy abstainers”, “moderate health risk behaviours” and “low risk drinkers but other health risk behaviours” classes, compared to those without depression. Women with probable anxiety showed 1.1 and 1.2 times greater odds of being in the “moderate health risk behaviours” and “low risk drinkers but other health risk behaviours” classes, respectively.

Both men and women reporting high strain (versus low strain) were significantly more likely to be in the “low risk drinkers but other health risk behaviours” class, but the odds ratios were small (1.1 for men, 1.2 for women).

## Discussion

### Key findings

This study utilised a large, representative sample of UK police employees, to explore how health risk behaviours cluster together and to determine their associations with mental health and job strain. Five classes were identified, with the smallest reflecting “high health risk behaviours” in both men and women, the most common reflecting “low risk drinkers but other health risk behaviours”, and two healthy classes, reflecting the “healthiest” and “healthy abstainers”. The final class for men reflected “some health risks but physically active” and “moderate health risk” for women. For both genders, those with probable depression, anxiety, or PTSD (compared to no mental health problem) had greater odds of being assigned to the “high health risk behaviours” class, though the odds were not as large for women. Men and women with probable depression were more likely to be in the “healthy abstainers” class, which was also apparent in men with probable anxiety and PTSD. Men and women reporting high strain (compared to low strain) had increased odds of being in the “low risk drinkers with other health risk behaviours” class. These findings highlight the importance of understanding clustering health risk behaviours, as the mental and physical health consequences will be greater for those with multiple risks [57].

### Classes of health risk behaviours

Previous literature has identified similar clusters of health risk behaviours, with clusters at either end of the spectrum (healthiest versus multiple health risk behaviours), with remaining classes reflecting a mixture of behaviours, which are usually more common [4, 12–14]. This is in line with the Theory of Triadic Influence (TTI), which proposes that behaviours are determined by one’s intentions, meaning health risk behaviours cluster together and healthy behaviours cluster together, because they align to similar social-cultural intentions [9]. Across the literature, there are differences in the proportion of participants considered “healthy”. In this study, 35% of male police employees and 42% of female police employees were in the two healthy classes (“healthiest” and “healthy abstainers”), compared to 80% of the Dutch general population [12], 23% of the UK general population [11], and 36% of men and 37% of women in the Australian general population [14], being in the “healthiest” classes. These findings suggest that UK police employees may engage in some health behaviours more than the UK general population, such as physical activity, but show other health risk behaviours, such as poor diet (13% met criteria for recommended fruit and vegetable intake, vs 25% of UK general population [58]). This may be because a certain level of fitness is required in police employees, and the latter may relate to shift work, which is associated with irregular eating patterns and unhealthy eating [59].

### Mental health and job strain

Previous findings of the same sample showed a relationship between poor mental health with harmful drinking and abstinence [25]. The present findings extend this, now showing a relationship between poor mental health and multiple health risk behaviours, with two–three times greater odds for male police employees with a mental health problem. This is in line with the few existing studies which demonstrate that individuals with poor mental health are more likely to be assigned to clusters of high health risk behaviours [12, 14, 60]. One study of vocational education students explored the clustering of physical and mental health risks, finding a class of high anxiety, high depression, and multiple health risk behaviours (smoking, risky alcohol use, poor diet, physical inactivity) [27]. A study independently examining associations between health risk behaviours and mental health, found that poor mental health was linked with smoking, low fruit and vegetable intake, and abstinence [61]. The latter is harmonious with current findings, showing poorer mental health in “healthy abstainers” than police employees in the “healthiest” class, and with a growing body of literature evidencing a J-shaped relationship, whereby poor mental health is associated with abstinence and heavy drinking [62–64]. This can be explained by the sick quitter hypothesis, which proposes that individuals become abstinent because of the physical or mental health consequences of heavy drinking [65]. It may be that those with poor mental health use health risk behaviours as a coping mechanism, such as smoking or heavy drinking, or health risk behaviours may be a consequence of having poor mental health, for example, not feeling motivated to exercise or eat healthily.

Regarding job strain, existing evidence in police employees showed that those reporting high strain (compared to low strain) had reduced odds of heavy drinking [25, 66], contradicting previous evidence [67]. The present findings can disentangle these unexpected findings, as police employees reporting high strain were more likely to be low risk drinkers but engage in other health risk behaviours. High demands and low control may reduce free time to eat healthily [45] or exercise regularly [68]. However, the current evidence-base is limited and mixed. Some research links high strain with smoking, and high control with high physical activity [69], with other research showing weak and inconsistent associations between high strain and health risk behaviours across samples [17]. Nevertheless, high strain is consistently associated with poor mental health [70] and increased days of sickness absence due to poor mental health [71]. In line with this, police employees in the “low risk drinkers but other health risk behaviours” class, characterised by high strain, also showed strong associations with probable depression and sickness absence, more so for men.

### Sociodemographic and occupational contributors

Male and female police employees engaging in multiple health risk behaviours were characterised by older age, lower educational attainment, having served longer in the police service, and having several days of sickness absence in the past year. These factors were previously found to be associated with harmful alcohol use in the same sample [25], and can now be linked to co-occurring health risk behaviours, such as smoking and poor diet. Conversely, police employees who were low risk drinkers but reported other health risk behaviours were characterised by younger age and fewer than five years in service, in line with findings from the UK general population, indicating a decline in youth drinking [72]. There were some gender differences in the characteristics of the classes. For example, having children was unrelated to class membership in men, but having no children (compared to one or two) was associated with moderate health risk behaviours in women. Compared to the healthiest class, all other classes were characterised by lower educational attainment. The link between low education and health risk behaviours is well-established [5, 73]. However, the specific pathways in which education influences health behaviours are complex and could be attributed to a range of economic and/or social inequalities [74].

### Strengths and limitations

This is the first study to determine classes of health risk behaviours in police employees, and one of few studies examining their mental health associations, in any population. This study utilised a representative sample with a good response rate and sufficient data to stratify by gender. Using LCA, this study determined co-occurring health risk behaviours and their associations, contrasting previous literature researching health risk behaviours independently [61]. However, these findings may not be the same for other occupational groups, given the specific nature of policing (e.g., expected level of physical fitness, time spent driving). Due to the cross-sectional nature of the data, temporal associations could not be determined, and it is unknown whether poor mental health is a contributor or consequence of engaging in health risk behaviours. Existing longitudinal evidence found associations with poor mental health and subsequent health risk behaviours but not vice versa [75], and this should be explored in police employees. This analysis is further limited, as the use of secondary data meant that only the available items could be included, and additional health risk behaviours (e.g., sleep quality) could not be explored.

### Implications

This study emphasises the importance of developing interventions which target co-occurring health risk behaviours, as individual behaviours have adverse physical and/or mental health consequences, but the combined impact of multiple health risk behaviours is much greater [57, 76, 77]. According to transfer theory, interventions should target co-occurring behaviours that are similar in their nature [78], as individuals are more likely to transfer their knowledge from one behaviour change to another if the behaviours share similar domains (relating to TTI [9]). Police employees experiencing poor mental health may engage in multiple health risk behaviours and workplace mental health support should incorporate a whole person approach, facilitating ones’ physical and mental health needs [79]. The workplace offers an advantageous environment for interventions targeting health risk behaviours, given the amount of time spent working [80, 81]. At an organisational level, health promotion campaigns can encourage healthy eating, smoking cessation, and physical activity. Addressing demand-control imbalances could allow more free time to for health behaviours. This study identified the characteristics of police employees who may be more likely to engage in multiple health risk behaviours and can be targeted by workplace interventions at an individual level.

## Conclusions

This is the first study to determine how health risk behaviours cluster in police employees, and their associations with mental health and job strain. Police employees with poor mental health were more likely to engage in multiple health risk behaviours, than those without a mental health problem. Those reporting high strain were more likely to be low risk drinkers but engage in other health risk behaviours, such as low physical activity and poor diet, than those reporting low strain. These findings highlight the importance of targeting co-occurring, not just individual, health risk behaviours to prevent physical and/or mental health consequences.

## Supplementary Information

**Additional file 1:** **Table S1. **Descriptive statistics for the sociodemographic and occupational variables (*N* = 40,986). **Table S2. **Multinomial logistic regressions exploring the sociodemographic and occupational associations with the identified classes of health (risk) behaviours for men (*N* = 25,788). Percentages are weighted with conditional probability weights. Unadjusted multinomial odds ratios (MOR) with 95% confidence intervals (CIs) are shown. **Table S3. **Multinomial logistic regressions exploring the sociodemographic and occupational associations with the identified classes of health (risk) behaviours for women (*N* = 15,198). Percentages are weighted with conditional probability weights. Unadjusted multinomial odds ratios (MOR) with 95% confidence intervals (CIs) are shown. **Fig S1.** Plot showing AIC, BIC, and SSABIC model fit criteria for each additional class.

## Data Availability

The data that support the findings of this study are available through a formal application process (https://police-health.org.uk/applying-access-resource), but restrictions apply to the availability of these data, which were used under license for the current study, and so are not publicly available.
