# Supplementary material for: A latent class analysis of health risk behaviours in the UK Police Service and their associations with mental health and job strain
Source: BMC Psychiatry. 2022 Jun 24;22:426. doi: 10.1186/s12888-022-04054-3 (PMC9233366; doi:10.1186/s12888-022-04054-3)
Supplement: Supplementary file 1 — Additional file 1: Table S1. Descriptive statistics for the sociodemographic and occupational variables (N = 40,986). Table S2. Multinomial logistic regressions exploring the sociodemographic and occupational associations with the identified classes of health (risk) behaviours for men (N = 25,788). Percentages are weighted with conditional probability weights. Unadjusted multinomial odds ratios (MOR) with 95% confidence intervals (CIs) are shown. Table S3. Multinomial logistic regressions exploring the sociodemographic and occupational associations with the identified classes of health (risk) behaviours for women (N = 15,198). Percentages are weighted with conditional probability weights. Unadjusted multinomial odds ratios (MOR) with 95% confidence intervals (CIs) are shown. Fig S1. Plot showing AIC, BIC, and SSABIC model fit criteria for each additional class. [file 12888_2022_4054_MOESM1_ESM.docx]

**Table S1.** Descriptive statistics for the sociodemographic and occupational variables (N = 40,986)

| **Variable** | | | **Complete N / % Missing** | **N** | **%** | **95% CI** |
| --- | --- | --- | --- | --- | --- | --- |
| *Sociodemographic variables* | | |  |  |  |  |
|  | Age | | 40,986 (0.00) |  |  |  |
|  |  | <29 |  | 5,653 | 13.79 | 13.46 to 14.13 |
|  |  | 30 to 39 |  | 13,547 | 33.05 | 32.60 to 33.51 |
|  |  | 40 to 49 |  | 15,919 | 38.84 | 38.37 to 39.31 |
|  |  | >50 |  | 5,867 | 14.31 | 13.98 to 14.66 |
|  | Gender | | 40,986 (0.00) |  |  |  |
|  |  | Men |  | 25,788 | 62.92 | 62.45 to 63.39 |
|  |  | Women |  | 15,198 | 37.08 | 36.61 to 37.55 |
|  | Country | | 40,236 (1.83) |  |  |  |
|  |  | England |  | 28,465 | 70.75 | 70.30 to 71.19 |
|  |  | Scotland |  | 6,428 | 15.98 | 15.62 to 16.34 |
|  |  | Wales |  | 5,343 | 13.28 | 12.95 to 13.61 |
|  | Educational attainment | | 40,828 (0.39) |  |  |  |
|  |  | GSCE/O-Level or below |  | 13,675 | 33.49 | 33.04 to 33.95 |
|  |  | A-levels/Highers or equivalent |  | 12,960 | 31.74 | 31.29 to 32.20 |
|  |  | Bachelor’s degree/Postgraduate |  | 11,326 | 27.74 | 27.31 to 28.18 |
|  |  | Vocational qualifications |  | 2,867 | 7.02 | 6.78 to 7.27 |
|  | Ethnicity ^a^ | | 40,786 (0.49) |  |  |  |
|  |  | White |  | 38,643 | 94.75 | 94.52 to 94.96 |
|  |  | Asian |  | 690 | 1.69 | 1.57 to 1.82 |
|  |  | Black |  | 438 | 1.07 | 0.98 to 1.18 |
|  |  | Mixed Ethnic Group |  | 456 | 1.12 | 1.02 to 1.22 |
|  |  | Other |  | 559 | 1.37 | 1.26 to 1.49 |
|  | Marital status | | 40,828 (0.39) |  |  |  |
|  |  | Married/Cohabiting |  | 31,710 | 77.67 | 77.26 to 78.07 |
|  |  | Divorced/Separated |  | 3,317 | 8.12 | 7.86 to 8.39 |
|  |  | Single |  | 4,854 | 11.89 | 11.58 to 12.21 |
|  |  | Other (e.g. widowed) |  | 947 | 2.32 | 2.18 to 2.47 |
|  | Children under 18 | | 40,828 (0.39) |  |  |  |
|  |  | 0 |  | 20,737 | 50.79 | 50.31 to 51.28 |
|  |  | 1 |  | 8,139 | 19.93 | 19.55 to 20.33 |
|  |  | 2 |  | 9,552 | 23.40 | 22.99 to 23.81 |
|  |  | 3 or more |  | 2,400 | 5.88 | 5.65 to 6.11 |
| *Occupational variables* | | |  |  |  |  |
|  | Police role | | 37,127 (9.42) |  |  |  |
|  |  | Police Officer |  | 26,031 | 70.11 | 69.65 to 70.58 |
|  |  | Police Staff |  | 10,361 | 27.91 | 27.45 to 28.37 |
|  |  | Other Ranks |  | 735 | 1.98 | 1.84 to 2.13 |
|  | Years in police force | | 40,931 (0.13) |  |  |  |
|  |  | Less than 5 |  | 8,466 | 20.68 | 20.29 to 21.08 |
|  |  | 6 to 10 |  | 9,231 | 22.55 | 22.15 to 22.96 |
|  |  | 11 to 20 |  | 12,679 | 30.98 | 30.53 to 31.43 |
|  |  | More than 20 |  | 10,555 | 25.79 | 25.37 to 26.21 |
|  | Income | | 40,828 (0.39) |  |  |  |
|  |  | Less than £25999 |  | 8,832 | 21.63 | 21.24 to 22.03 |
|  |  | £26000 - £37999 |  | 16,820 | 41.20 | 40.72 to 41.68 |
|  |  | £38000 - £59999 |  | 13,869 | 33.97 | 33.51 to 34.43 |
|  |  | More than £60000 |  | 1,307 | 3.20 | 3.03 to 3.38 |
|  | Days of sickness absence in past year | | 40,928 (0.14) |  |  |  |
|  |  | None |  | 18,956 | 46.32 | 45.83 to 46.80 |
|  |  | 1 to 5 |  | 13,504 | 32.99 | 32.54 to 33.45 |
|  |  | 6 to 10 |  | 3,703 | 9.05 | 8.77 to 9.33 |
|  |  | More than 10 |  | 4,765 | 11.64 | 11.34 to 11.96 |

^a^ White (White British, White Irish, any other White background), Asian (Asian Indian, Asian Bangladeshi, Asian Pakistani, Chinese, any other Asian background), Black (Black African, Black Caribbean, any other Black background), Mixed Ethnic Group (White and Asian, White and Black African, White and Black Caribbean, any other mixed ethnic group).

**Table S2.** Multinomial logistic regressions exploring the sociodemographic and occupational associations with the identified classes of health (risk) behaviours for men (N = 25,788). Percentages are weighted with conditional probability weights. Unadjusted multinomial odds ratios (MOR) with 95% confidence intervals (CIs) are shown.

|  | | **Class 1 N = 5,307**  **Healthiest (Ref.)** | **Class 2 N = 3,560**  **Healthy abstainers** | | **Class 3 N = 7,092**  **Health risks but physically active** | | **Class 4 N = 8,839**  **Low risk drinkers but other risks** | | **Class 5 N = 990**  **High health risk behaviours** | |
| --- | --- | --- | --- | --- | --- | --- | --- | --- | --- | --- |
| **Men (N = 25,788)** | | **N (%)** | **N (%)** | **MOR (95% CI)** | **N (%)** | **MOR (95% CI)** | **N (%)** | **MOR (95% CI)** | **N (%)** | **MOR (95% CI)** |
| Age | |  |  |  |  |  |  |  |  |  |
|  | <29 | 601 (11.88) | 309 (9.74) | 1.00 | 719 (10.22) | 1.00 | 1,158 (13.85) | 1.00 | 65 (6.36) | 1.00 |
|  | 30 to 39 | 1,755 (33.58) | 1,044 (30.73) | 1.12 (0.95 to 1.31) | 2,168 (30.65) | 1.06 (0.93 to 1.21) | 2,946 (33.87) | 0.86 (0.77 to 0.97)* | 274 (27.67) | 1.54 (1.15 to 2.05)** |
|  | 40 to 49 | 2,137 (39.76) | 1,479 (40.81) | 1.25 (1.07 to 1.46)** | 3,202 (45.14) | 1.32 (1.17 to 1.49)*** | 3,574 (39.98) | 0.86 (0.77 to 0.97)* | 468 (47.55) | 2.23 (1.69 to 2.95)*** |
|  | >50 | 814 (14.78) | 728 (18.72) | 1.54 (1.30 to 1.84)*** | 1,003 (14.00) | 1.10 (0.95 to 1.27) | 1,161 (12.29) | 0.71 (0.62 to 0.82)*** | 183 (18.42) | 2.33 (1.71 to 3.16)*** |
| Country | |  |  |  |  |  |  |  |  |  |
|  | England | 3,695 (70.56) | 2,701 (76.65) | 1.00 | 4,666 (66.86) | 1.00 | 6,077 (69.66) | 1.00 | 662 (68.01) | 1.00 |
|  | Scotland | 810 (15.83) | 450 (13.39) | 0.78 (0.68 to 0.89)*** | 1,299 (18.86) | 1.26 (1.14 to 1.39)*** | 1,638 (19.34) | 1.24 (1.12 to 1.36)*** | 145 (14.95) | 0.98 (0.80 to 1.19) |
|  | Wales | 702 (13.60) | 344 (9.97) | 0.67 (0.58 to 0.79)*** | 965 (14.28) | 1.11 (0.99 to 1.23) | 966 (11.00) | 0.82 (0.74 to 0.91)*** | 165 (17.05) | 1.30 (1.07 to 1.57)** |
| Education | |  |  |  |  |  |  |  |  |  |
|  | GSCE/O-Level or below | 1,665 (35.16) | 1,309 (36.56) | 1.00 | 2,479 (35.16) | 1.00 | 3,120 (35.05) | 1.00 | 433 (43.82) | 1.00 |
|  | Vocational qualifications | 374 (7.17) | 249 (7.17) | 0.85 (0.71 to 1.03) | 484 (6.84) | 0.85 (0.73 to 0.99)* | 637 (7.32) | 0.91 (0.79 to 1.05) | 52 (5.30) | 0.53 (0.39 to 0.72)*** |
|  | A-levels/Highers | 1,709 (32.21) | 1,073 (30.53) | 0.81 (0.73 to 0.90)*** | 2,287 (32.44) | 0.89 (0.82 to 0.98)* | 2,887 (33.28) | 0.92 (0.84 to 1.00) | 293 (30.25) | 0.67 (0.57 to 0.79)*** |
|  | Bachelor’s/Postgraduate | 1,551 (29.44) | 914 (25.73) | 0.75 (0.67 to 0.84)*** | 1,811 (25.56) | 0.77 (0.70 to 0.85)*** | 2,146 (24.35) | 0.74 (0.67 to 0.81)*** | 206 (20.63) | 0.50 (0.42 to 0.60)*** |
| Ethnicity | |  |  |  |  |  |  |  |  |  |
|  | White | 5,046 (95.41) | 3,087 (85.46) | 1.00 | 6,809 (96.59) | 1.00 | 8,359 (95.13) | 1.00 | 956 (97.11) | 1.00 |
|  | Asian | 49 (1.00) | 254 (8.49) | 9.47 (6.90 to 12.99)*** | 45 (0.62) | 0.62 (0.41 to 0.93)* | 87 (1.02) | 1.02 (0.71 to 1.47) | 5 (0.45) | 0.44 (0.17 to 1.15) |
|  | Black | 46 (0.85) | 74 (2.32) | 3.04 (2.08 to 4.46)*** | 16 (0.22) | 0.26 (0.14 to 0.46)*** | 77 (0.86) | 1.01 (0.69 to 1.47) | 0 (0.00) | - |
|  | Mixed Race | 61 (1.20) | 39 (1.15) | 1.07 (0.71 to 1.62) | 72 (1.01) | 0.83 (0.59 to 1.18) | 113 (1.29) | 1.08 (0.78 to 1.49) | 8 (0.79) | 0.65 (0.31 to 1.38) |
|  | Other | 83 (1.54) | 80 (2.59) | 1.88 (1.37 to 2.58)*** | 112 (1.55) | 0.99 (0.74 to 1.33) | 148 (1.70) | 1.11 (0.84 to 1.47) | 16 (1.46) | 1.05 (0.61 to 1.81) |
| Marital status | |  |  |  |  |  |  |  |  |  |
|  | Married/Cohabiting | 4,510 (84.74) | 3,004 (83.58) | 1.00 | 5,803 (82.08) | 1.00 | 7,339 (83.07) | 1.00 | 824 (84.12) | 1.00 |
|  | Divorced/Separated | 318 (5.98) | 207 (5.95) | 1.01 (0.84 to 1.22) | 532 (7.54) | 1.30 (1.12 to 1.51)*** | 610 (6.98) | 1.19 (1.03 to 1.38)* | 81 (8.17) | 1.38 (1.06 to 1.78)* |
|  | Single | 370 (7.24) | 284 (8.95) | 1.25 (1.06 to 1.48)** | 606 (8.68) | 1.24 (1.08 to 1.42)** | 684 (8.09) | 1.14 (1.00 to 1.31) | 72 (6.98) | 0.97 (0.74 to 1.27) |
|  | Other | 101 (2.04) | 50 (1.52) | 0.75 (0.53 to 1.07) | 120 (1.70) | 0.86 (0.65 to 1.13) | 157 (1.86) | 0.93 (0.72 to 1.20) | 7 (0.73) | 0.36 (0.17 to 0.79)* |
| Children under 18 | |  |  |  |  |  |  |  |  |  |
|  | 0 | 2,324 (44.03) | 1,555 (44.15) | 1.00 | 3,325 (47.23) | 1.00 | 3,876 (44.05) | 1.00 | 453 (45.56) | 1.00 |
|  | 1 | 1,112 (20.99) | 741 (20.96) | 1.00 (0.89 to 1.12) | 1,407 (19.87) | 0.88 (0.80 to 0.97)* | 1,938 (22.14) | 1.05 (0.96 to 1.16) | 178 (18.33) | 0.84 (0.70 to 1.02) |
|  | 2 | 1,467 (27.57) | 962 (26.67) | 0.98 (0.87 to 1.07) | 1,839 (25.92) | 0.88 (0.80 to 0.96)** | 2,360 (26.82) | 0.97 (0.89 to 1.06) | 272 (27.87) | 0.98 (0.83 to 1.15) |
|  | 3 or more | 396 (7.41) | 287 (8.22) | 1.07 (0.93 to 1.31) | 490 (6.98) | 0.88 (0.76 to 1.02) | 616 (6.99) | 0.94 (0.82 to 1.08) | 81 (8.24) | 1.07 (0.83 to 1.39) |
| Police role | |  |  |  |  |  |  |  |  |  |
|  | Police Officer | 3,881 (82.04) | 2,380 (73.87) | 1.00 | 5,454 (83.90) | 1.00 | 6,718 (83.97) | 1.00 | 775 (84.22) | 1.00 |
|  | Police Staff | 790 (16.36) | 789 (24.38) | 1.66 (1.48 to 1.86)*** | 944 (14.65) | 0.88 (0.79 to 0.97)* | 1,190 (14.58) | 0.87 (0.79 to 0.96)* | 128 (13.86) | 0.83 (0.69 to 1.01) |
|  | Other Ranks | 76 (1.45) | 54 (1.75) | 1.21 (0.84 to 1.86) | 95 (1.45) | 0.88 (0.65 to 1.21) | 120 (1.45) | 0.89 (0.66 to 1.19) | 18 (1.93) | 1.17 (0.69 to 1.98) |
| Years in police force | |  |  |  |  |  |  |  |  |  |
|  | Less than 5 | 851 (16.53) | 543 (16.27) | 1.00 | 1,010 (14.46) | 1.00 | 1,574 (18.43) | 1.00 | 85 (8.39) | 1.00 |
|  | 6 to 10 | 1,079 (20.73) | 860 (25.11) | 1.23 (1.07 to 1.42)** | 1,287 (18.14) | 1.00 (0.88 to 1.13) | 1,962 (22.66) | 0.98 (0.87 to 1.10) | 168 (16.98) | 1.61 (1.22 to 2.13)*** |
|  | 11 to 20 | 1,716 (32.33) | 1,083 (30.72) | 0.97 (0.84 to 1.11) | 2,217 (32.66) | 1.15 (1.03 to 1.29** | 2,789 (31.63) | 0.88 (0.79 to 0.97)* | 340 (34.25) | 2.09 (1.62 to 2.69)*** |
|  | More than 20 | 1,650 (30.41) | 1,064 (27.90) | 0.93 (0.81 to 1.07) | 2,463 (34.74) | 1.31 (1.17 to 1.46)*** | 2,508 (27.28) | 0.80 (0.72 to 0.90)*** | 397 (40.38) | 2.62 (2.03 to 3.37)*** |
| Income | |  |  |  |  |  |  |  |  |  |
|  | Less than £25999 | 494 (9.30) | 466 (13.34) | 1.00 | 573 (8.24) | 1.00 | 835 (9.71) | 1.00 | 60 (5.97) | 1.00 |
|  | £26000 - £37999 | 2,162 (41.17) | 1,481 (42.43) | 0.72 (0.62 to 0.83)*** | 2,794 (39.52) | 1.08 (0.95 to 1.24) | 3,883 (44.53) | 1.04 (0.91 to 1.18) | 388 (39.75) | 1.50 (1.12 to 2.02)** |
|  | £38000 - £59999 | 2,400 (44.95) | 1,462 (40.67) | 0.63 (0.54 to 0.73)*** | 3,332 (47.16) | 1.18 (1.04 to 1.35)* | 3,733 (42.09) | 0.90 (0.79 to 1.02) | 496 (50.36) | 1.74 (1.31 to 2.33)*** |
|  | More than £60000 | 243 (4.58) | 136 (3.92) | 0.54 (0.42 to 0.70)*** | 362 (5.08) | 1.25 (1.02 to 1.54)* | 339 (3.66) | 0.77 (0.62 to 0.94)** | 40 (3.66) | 1.33 (0.86 to 2.06) |
| Days of sickness absence in past year | |  |  |  |  |  |  |  |  |  |
|  | None | 2,880 (54.30) | 1,777 (40.96) | 1.00 | 3,631 (51.26) | 1.00 | 4,458 (50.52) | 1.00 | 491 (49.75) | 1.00 |
|  | 1 to 5 | 1,573 (29.81) | 1,069 (29.56) | 1.08 (0.97 to 1.19) | 2,197 (31.07) | 1.10 (1.02 to 1.20)* | 2,780 (31.62) | 1.14 (1.05 to 1.23)** | 277 (27.93) | 1.02 (0.87 to 1.20) |
|  | 6 to 10 | 386 (7.16) | 305 (8.76) | 1.33 (1.12 to 1.57)** | 563 (7.96) | 1.18 (1.02 to 1.36)* | 719 (8.18) | 1.23 (1.07 to 1.41)** | 85 (8.87) | 1.35 (1.05 to 1.75)** |
|  | More than 10 | 468 (8.73) | 405 (11.72) | 1.46 (1.26 to 1.70)*** | 686 (9.71) | 1.19 (1.04 to 1.34)* | 862 (9.68) | 1.19 (1.05 to 1.35)** | 134 (13.45) | 1.68 (1.35 to 2.09)*** |

***p<0.001, **p<0.01, *p<0.05

**Table S3.** Multinomial logistic regressions exploring the sociodemographic and occupational associations with the identified classes of health (risk) behaviours for women (N = 15,198). Percentages are weighted with conditional probability weights. Unadjusted multinomial odds ratios (MOR) with 95% confidence intervals (CIs) are shown.

|  | | **Class 1 N = 4,543**  **Healthiest (Ref)** | **Class 2 N = 1,894**  **Healthy abstainers** | | **Class 3 N = 2,072**  **Moderate health risk behaviours** | | **Class 4 N = 6,121**  **Low risk drinkers but other risks** | | **Class 5 N = 568**  **High health risk behaviours** | |
| --- | --- | --- | --- | --- | --- | --- | --- | --- | --- | --- |
| **Women (N =15,198)** | | **N (%)** | **N (%)** | **MOR (95% CI)** | **N (%)** | **MOR (95% CI)** | **N (%)** | **MOR (95% CI)** | **N (%)** | **MOR (95% CI)** |
| Age | |  |  |  |  |  |  |  |  |  |
|  | <29 | 732 (16.28) | 304 (16.17) | 1.00 | 400 (20.15) | 1.00 | 1,306 (22.22) | 1.00 | 59 (10.13) | 1.00 |
|  | 30 to 39 | 1,484 (32.91) | 716 (37.85) | 1.16 (0.98 to 1.36) | 691 (33.63) | 0.83 (0.71 to 0.96)* | 2,283 (37.67) | 0.84 (0.75 to 0.94)** | 186 (37.85) | 1.58 (1.15 to 2.17)** |
|  | 40 to 49 | 1,531 (33.62) | 576 (30.24) | 0.91 (0.77 to 1.07) | 760 (36.08) | 0.87 (0.74 to 1.01) | 1,937 (30.97) | 0.67 (0.60 to 0.76)*** | 255 (45.55) | 2.18 (1.60 to 2.95)*** |
|  | >50 | 796 (17.19) | 298 (15.73) | 0.92 (0.76 to 1.11) | 221 (10.13) | 0.48 (0.39 to 0.58)*** | 595 (9.15) | 0.39 (0.34 to 0.45)*** | 68 (11.96) | 1.12 (0.77 to 1.62) |
| Country | |  |  |  |  |  |  |  |  |  |
|  | England | 3,197 (71.75) | 1,431 (76.79) | 1.00 | 1,398 (68.11) | 1.00 | 4,234 (70.00) | 1.00 | 404 (72.91) | 1.00 |
|  | Scotland | 583 (13.21) | 187 (10.16) | 0.72 (0.60 to 0.86)*** | 309 (15.43) | 1.23 (1.05 to 1.44)* | 943 (15.89) | 1.23 (1.10 to 1.38)*** | 64 (11.33) | 0.84 (0.63 to 1.12) |
|  | Wales | 678 (15.04) | 240 (13.05) | 0.81 (0.69 to 0.95)* | 330 (16.47) | 1.15 (0.99 to 1.34) | 834 (14.11) | 0.96 (0.86 to 1.08) | 89 (15.76) | 1.03 (0.80 to 1.33) |
| Education | |  |  |  |  |  |  |  |  |  |
|  | GSCE/O-Level or below | 1,303 (28.60) | 633 (33.42) | 1.00 | 635 (30.14) | 1.00 | 1,892 (30.60) | 1.00 | 206 (36.76) | 1.00 |
|  | Vocational qualifications | 296 (6.53) | 146 (7.87) | 1.03 (0.83 to 1.29) | 145 (7.06) | 1.03 (0.82 to 1.28) | 447 (7.41) | 1.06 (0.90 to 1.25) | 37 (6.72) | 0.80 (0.55 to 1.17) |
|  | A-levels/Highers | 1,381 (30.51) | 569 (30.02) | 0.84 (0.73 to 0.97) | 629 (30.53) | 0.95 (0.83 to 1.09) | 1,944 (32.01) | 0.98 (0.89 to 1.08) | 188 (33.10) | 0.84 (0.68 to 1.05) |
|  | Bachelor’s/Postgraduate | 1,548 (34.37) | 540 (28.69) | 0.71 (0.62 to 0.82) | 655 (32.28) | 0.89 (0.78 to 1.02) | 1,818 (29.98) | 0.82 (0.74 to 0.90)*** | 137 (23.42) | 0.53 (0.42 to 0.67)*** |
| Ethnicity | |  |  |  |  |  |  |  |  |  |
|  | White | 4,333 (95.89) | 1,642 (86.80) | 1.00 | 2,037 (98.61) | 1.00 | 5,823 (95.50) | 1.00 | 551 (97.15) | 1.00 |
|  | Asian | 53 (1.17) | 107 (5.72) | 5.38 (3.84 to 7.54)*** | 3 (0.14) | 0.11 (0.04 to 0.38)*** | 84 (1.35) | 1.15 (0.81 to 1.64) | 3 (0.56) | 0.47 (0.14 to 1.54) |
|  | Black | 50 (1.06) | 81 (4.41) | 4.61 (3.21 to 6.60)*** | 7 (0.32) | 0.30 (0.13 to 0.67)** | 83 (1.33) | 1.26 (0.88 to 1.80) | 4 (0.71) | 0.66 (0.23 to 1.91) |
|  | Mixed Race | 46 (1.01) | 30 (1.60) | 1.75 (1.10 to 2.80)* | 12 (0.54) | 0.52 (0.27 to 1.00)* | 69 (1.11) | 1.11 (0.76 to 1.62) | 6 (1.12) | 1.09 (0.45 to 2.63) |
|  | Other | 40 (0.87) | 27 (1.47) | 1.86 (1.14 to 3.05)* | 8 (0.38) | 0.43 (0.20 to 0.93)* | 42 (0.71) | 0.81 (0.52 to 1.26) | 3 (0.47) | 0.53 (0.15 to 1.83) |
| Marital status | |  |  |  |  |  |  |  |  |  |
|  | Married/Cohabiting | 3,164 (69.78) | 1,317 (69.85) | 1.00 | 1,320 (63.38) | 1.00 | 4,059 (66.26) | 1.00 | 370 (64.73) | 1.00 |
|  | Divorced/Separated | 470 (10.27) | 178 (9.47) | 0.92 (0.77 to 1.10) | 222 (10.68) | 1.15 (0.96 to 1.37) | 622 (10.17) | 1.03 (0.91 to 1.19) | 77 (13.67) | 1.43 (1.09 to 1.88)** |
|  | Single | 751 (16.75) | 337 (17.70) | 1.06 (0.91 to 1.22) | 430 (21.50) | 1.41 (1.23 to 1.62)*** | 1,222 (20.34) | 1.28 (1.15 to 1.42)*** | 98 (17.67) | 1.14 (0.89 to 1.45) |
|  | Other | 143 (3.20) | 56 (3.23) | 0.93 (0.68 to 1.28) | 92 (4.43) | 1.52 (1.16 to 2.01)** | 198 (3.23) | 1.06 (0.85 to 1.33) | 23 (3.93) | 1.32 (0.83 to 2.11) |
| Children under 18 | |  |  |  |  |  |  |  |  |  |
|  | 0 | 2,780 (61.55) | 1,087 (57.48) | 1.00 | 1,361 (66.66) | 1.00 | 3,625 (59.53) | 1.00 | 351 (62.12) | 1.00 |
|  | 1 | 789 (17.26) | 400 (21.36) | 1.32 (1.15 to 1.52)*** | 288 (13.69) | 0.73 (0.63 to 0.85)*** | 1,193 (19.65) | 1.18 (1.06 to 1.31)** | 93 (16.13) | 0.93 (0.72 to 1.19) |
|  | 2 | 808 (17.83) | 332 (17.43) | 1.05 (0.90 to 1.21) | 346 (16.13) | 0.84 (0.73 to 0.98)* | 1,064 (17.43) | 1.00 (0.90 to 1.12) | 102 (18.08) | 1.00 (0.79 to 1.28) |
|  | 3 or more | 151 (3.35) | 69 (3.72) | 1.19 (0.89 to 1.60) | 69 (3.35) | 0.92 (0.68 to 1.24) | 219 (3.49) | 1.08 (0.87 to 1.34) | 22 (3.67) | 1.08 (0.67 to 1.75) |
| Police role | |  |  |  |  |  |  |  |  |  |
|  | Police Officer | 1,976 (49.11) | 757 (44.42) | 1.00 | 1,013 (54.70) | 1.00 | 2,802 (50.14) | 1.00 | 275 (54.69) | 1.00 |
|  | Police Staff | 1,971 (47.85) | 885 (52.06) | 1.20 (1.07 to 1.35)** | 802 (42.76) | 0.80 (0.72 to 0.90)*** | 2,640 (47.58) | 0.97 (0.90 to 1.06) | 222 (42.63) | 0.80 (0.66 to 0.97)* |
|  | Other Ranks | 122 (3.04) | 59 (3.52) | 1.28 (0.93 to 1.77) | 49 (2.55) | 0.75 (0.53 to 1.07) | 127 (2.28) | 0.73 (0.57 to 0.95)* | 15 (2.67) | 0.79 (0.45 to 1.39) |
| Years in police force | |  |  |  |  |  |  |  |  |  |
|  | Less than 5 | 1,238 (27.37) | 515 (27.06) | 1.00 | 593 (29.13) | 1.00 | 1,938 (32.41) | 1.00 | 119 (20.32) | 1.00 |
|  | 6 to 10 | 1,146 (25.41) | 533 (28.32) | 1.13 (0.97 to 1.31) | 453 (21.97) | 0.81 (0.70 to 0.94)** | 1,627 (26.67) | 0.89 (0.80 to 1.00)* | 116 (20.28) | 1.08 (0.82 to 1.42) |
|  | 11 to 20 | 1,346 (29.58) | 547 (28.94) | 0.99 (0.86 to 1.14) | 654 (31.70) | 1.01 (0.88 to 1.16) | 1,681 (27.19) | 0.78 (0.71 to 0.86)*** | 206 (36.50) | 1.66 (1.30 to 2.13)*** |
|  | More than 20 | 809 (17.64) | 296 (15.67) | 0.90 (0.76 to 1.07) | 371 (17.19) | 0.92 (0.78 to 1.08) | 870 (13.73) | 0.66 (0.58 to 0.74)*** | 127 (22.91) | 1.75 (1.33 to 2.30)\*** |
| Income | |  |  |  |  |  |  |  |  |  |
|  | Less than £25999 | 1,939 (42.61) | 847 (44.65) | 1.00 | 790 (38.08) | 1.00 | 2,647 (43.31) | 1.00 | 181 (31.11) | 1.00 |
|  | £26000 - £37999 | 1,784 (39.48) | 755 (40.25) | 0.97 (0.86 to 1.10) | 816 (39.85) | 1.13 (1.00 to 1.27)* | 2,511 (41.70) | 1.04 (0.95 to 1.13) | 246 (44.09) | 1.53 (1.24 to 1.88)*** |
|  | £38000 - £59999 | 743 (16.54) | 266 (14.04) | 0.81 (0.69 to 0.95)* | 423 (20.48) | 1.38 (1.19 to 1.61)*** | 878 (13.96) | 0.83 (0.74 to 0.93)** | 136 (23.98) | 1.98 (1.55 to 2.54)*** |
|  | More than £60000 | 62 (1.59) | 20 (1.06) | 0.74 (0.44 to 1.23) | 35 (1.59) | 1.30 (0.84 to 2.00) | 65 (1.04) | 0.74 (0.52 to 1.07) | 5 (0.82) | 0.82 (0.32 to 2.09) |
| Days of sickness absence in past year | |  |  |  |  |  |  |  |  |  |
|  | None | 1,829 (40.42) | 674 (35.41) | 1.00 | 748 (36.39) | 1.00 | 2,271 (36.83) | 1.00 | 197 (34.78) | 1.00 |
|  | 1 to 5 | 1,614 (35.55) | 648 (34.23) | 1.10 (0.97 to 1.25) | 791 (38.25) | 1.20 (1.06 to 1.35)** | 2,363 (38.86) | 1.20 (1.10 to 1.31)*** | 192 (33.72) | 1.10 (0.89 to 1.37) |
|  | 6 to 10 | 475 (10.37) | 207 (10.97) | 1.21 (1.00 to 1.46)* | 227 (10.96) | 1.17 (0.98 to 1.41) | 664 (10.96) | 1.16 (1.01 to 1.33)* | 72 (12.92) | 1.45 (1.08 to 1.95)** |
|  | More than 10 | 623 (13.66) | 364 (19.39) | 1.62 (1.38 to 1.90)*** | 299 (14.40) | 1.17 (0.99 to 1.38) | 817 (13.35) | 1.07 (0.95 to 1.21) | 107 (18.58) | 1.58 (1.22 to 2.05)** |

***p<0.001, **p<0.01, *p<0.05

*Figure S1.* Plot showing AIC, BIC, and SSABIC model fit criteria for each additional class.
